# Paraoxonases-2 and -3 Are Important Defense Enzymes against *Pseudomonas aeruginosa* Virulence Factors due to Their Anti-Oxidative and Anti-Inflammatory Properties

**DOI:** 10.1155/2012/352857

**Published:** 2012-04-12

**Authors:** Eva-Maria Schweikert, Julianna Amort, Petra Wilgenbus, Ulrich Förstermann, John F. Teiber, Sven Horke

**Affiliations:** ^1^Institute of Pharmacology, University Medical Center of the Johannes Gutenberg-University Mainz, Obere Zahlbacher Straße 67, 55131 Mainz, Germany; ^2^Division of Epidemiology, Department of Internal Medicine, The University of Texas Southwestern Medical Center, 5323 Harry Hines Boulevard, Dallas, TX 75390, USA

## Abstract

The pathogen *Pseudomonas aeruginosa* causes serious damage in immunocompromised patients by secretion of various virulence factors, among them the quorum sensing N-(3-oxododecanoyl)-L-homoserine lactone (3OC12) and the redox-active pyocyanin (PCN). Paraoxonase-2 (PON2) may protect against *P. aeruginosa* infections, as it efficiently inactivates 3OC12 and diminishes PCN-induced oxidative stress. This defense could be circumvented because 3OC12 mediates intracellular Ca^2+^-rise in host cells, which causes rapid inactivation and degradation of PON2. Importantly, we recently found that the PON2 paralogue PON3 prevents mitochondrial radical formation. Here we investigated its role as additional potential defense mechanism against *P. aeruginosa* infections. Our studies demonstrate that PON3 diminished PCN-induced oxidative stress. Moreover, it showed clear anti-inflammatory potential by protecting against NF-*κ*B activation and IL-8 release. The latter similarly applied to PON2. Furthermore, we observed a Ca^2+^-mediated inactivation and degradation of PON3, again in accordance with previous findings for PON2. Our results suggest that the anti-oxidative and anti-inflammatory functions of PON2 and PON3 are an important part of our innate defense system against *P. aeruginosa* infections. Furthermore, we conclude that *P. aeruginosa* circumvents PON3 protection by the same pathway as for PON2. This may help identifying underlying mechanisms in order to sustain the protection afforded by these enzymes.

## 1. Introduction

 The bacterium *Pseudomonas aeruginosa* is an opportunistic nosocomial pathogen, which infects the pulmonary tract of, for example, immunocompromised patients or those suffering from cystic fibrosis, pneumonia, burn wounds, HIV, or cancer chemotherapy [1]. The infection causes serious damage in the host, complicated by an often hindered antibiotic treatment due to multiresistances and biofilm formation that provides physical protection. Furthermore, *P. aeruginosa *secrets a variety of virulence factors to regulate bacterial communication and weaken the defense mechanisms of the infected host. Two important factors are the quorum sensing signal *N*-(3-oxododecanoyl)-L-homoserine lactone (3OC12) and the redox-active pyocyanin (PCN). 3OC12 is a mediator of the cell-density-dependent signaling system known as quorum sensing, by which the bacteria coordinate their gene expression. If bacterial density and 3OC12 concentration exceed a certain threshold, the bacteria become virulent by expression of virulence factors (immunogenic exoenzymes and toxins) and by inducing inflammation. The lactone 3OC12 has numerous immunomodulatory and inflammatory properties, such as an inhibitory effect on dendritic cells and T-cell activation [2], proinflammatory induction of IL-6 and IL-8 in airway epithelial cells and lung fibroblasts [3], and promoting apoptosis [4, 5]. Studies in mice have shown that 3OC12 is a critical determinant for bacterial colocalization and the establishment of chronic lung infections [6]. Therefore, the development of quorum sensing inhibitors would be a major advance in the ability to combat *P. aeruginosa* infections [7, 8].

 The production of the virulence factor PCN is positively regulated by quorum sensing signals including 3OC12 [9]. PCN causes oxidative stress and has a broad range of effects on airway epithelial cells such as cellular senescence and ciliary dyskinesia, induction of IL-8 secretion, decrease of glutathione levels, and inhibition of catalase activity [9, 10]. The redox-activity of PCN is central to the damage observed in exposed host cells. The zwitterionic PCN transfers electrons from reduced NADH or NADPH in the cytosol to molecular oxygen leading to production of superoxide (O_2_^−^), which is converted to H_2_O_2_ [11]. Additionally, PCN causes a disturbance of the antimicrobial Duox/SCN^−^/LPO-system, by consuming the same substrates (molecular oxygen and NADPH) [12]. The requirement for PCN in lung infection was demonstrated in an acute pneumonia mice model: *P. aeruginosa* strains lacking the ability to produce PCN are much more rapidly cleared from lungs and showed less virulence than the wild-type strain [13].

 The paraoxonase family consists of the three members PON1, PON2, and PON3, which exhibit about 70% similarity at the amino acid level [14]. PON1 is associated with HDL in serum, whereas PON2 and PON3 are intracellular proteins. In contrast to PON2, which is ubiquitously expressed, PON3 appears restricted to fewer tissues/cells; its expression in cells relevant to cardiovascular diseases is contradictory, because Marsillach et al. [15] found PON3 by immunohistochemistry in human vascular walls and macrophages, while our studies revealed absence in macrophages, endothelial, smooth muscle, and many other cell types [16]. All three PONs share a lactonase activity with distinct and overlapping substrate specificities [17–19]. PON2 dominantly hydrolyzes 3OC12 presumably resulting in the ability to interfere with quorum sensing, which may significantly attenuate bacterial virulence of *P. aeruginosa*. In support of this concept, epithelial tracheal cells from PON2 deficient mice showed a reduced ability to inactivate 3OC12 [20]. In addition to its lactonase activity, PON2 is a major anti-oxidative protein that diminishes mitochondrial superoxide production and thus considerably determines cell survival [21–23]. In particular, it has also been shown that PON2 diminishes PCN-induced ROS production in human epithelial cells [24]. Intriguingly, 3OC12 causes a rapid Ca^2+^-mediated PON2 inactivation and degradation in cultured cells by a yet unknown mechanism, which enables 3OC12 to protect itself from its hydrolysis by PON2 [25]. As a consequence, 3OC12 potentiates the formation of ROS induced by PCN, revealing a potential mechanism by which the bacterium may circumvent the protection afforded by PON2 [25].

 Our recent studies demonstrated that PON3, much like PON2, reduced the generation of mitochondrial superoxide. We also revealed that PON3 was frequently found overexpressed in tumors. There, it reduced susceptibility to chemotherapeutics and reduced apoptosis, in line with the central involvement of mitochondrial ROS to cell death [16]. Hence, we hypothesized that PON3 attenuated PCN-induced oxidative stress, which was tested here for the first time. Our studies also included inflammatory pathways subsequent to PCN stimulation, that is, NF-*κ*B activation and secretion of various cytokines. Our results support the concept of marked anti-inflammatory roles for PON2 and PON3. Finally, we found that both enzymes, PON2 and PON3, were inactivated and degraded in response to Ca^2+^-disturbances caused by 3OC12. Identifying the underlying pathway(s) by which the PONs are downregulated may reveal a therapeutic target, which could be exploited to help sustain or enhance the host's PON2 and PON3 activities. Such a clinical intervention could be of great benefit in the defense against *P. aeruginosa* infections.

## 2. Material and Methods

### 2.1. Cell Culture and Material

Human endothelial EA.hy 926 cells obtained from the ATCC were cultured in Dulbecco's modified Eagle's medium without Phenol Red (Sigma, St. Louis, MO, USA) containing sodium pyruvate (PAA Laboratories, Pasching, Austria), antibiotics penicillin/streptomycin, hypoxanthine/aminopterin/thymidine supplement, L-Glutamine (Invitrogen, Carlsbad, CA, USA), and 10% (v/v) fetal calf serum (PAA). Human PON2 or PON3 cDNA was subcloned into pDsRed-Express-N1 or pEGFP-N1 plasmids (Clontech). Stable cell lines, plasmids, and transfection procedures were described before [25, 26]. HEK293 and A549 cells were from the German Collection of Microorganisms and Cell Cultures. HEK293 received the same medium as EA.hy 926, but without hypoxanthine/aminopterin/thymidine supplement. A549 cells received the same medium as HEK293, but 5% serum. Cells were cultured at 37°C in a humidified atmosphere with 5% CO_2_ (10% for EA.hy 926). Pyocyanin was purchased from Cayman Chemical Company (Ann Arbor, MI, USA); Mito-HE and thapsigargin were from Molecular Probes; L-012 was from Wako Chemicals (Neuss, Germany), and all other reagents were from Sigma.

### 2.2. ELISA

EA.hy 926 cells were seeded one day prior to stimulation in 6-well dishes at 80% confluency (5 × 10^5^/well). Treatment occurred in 1.5 mL medium without FCS for 16 h with or without pyocyanin (10 *μ*M). Cell supernatants were taken for Multi-Analyte ELISArray Kit for human inflammatory cytokines (SA Biosciences, Frederick, MD, USA) according to the supplier's instructions. Absorbance was determined using a FluoStar Optima microplate reader (BMG Labtechnologies). Corrected OD was calculated by subtracting the A450 reading by the A570 reading to clear any minor optical imperfections in the ELISA plate.

### 2.3. Reporter Gene Assays

 Cells at 80% confluency were cotransfected with pcDNA3-HA or pcDNA3-PON2-HA or pEGFP-N1 or pEGFP-N1-PON3 and a plasmid allowing for constitutive renilla luciferase expression (a kind gift of H. Kleinert, University Medical Centre, Mainz) and the NF-*κ*B reporter plasmid (pGL4.32[luc2P/NF-*κ*B-RE/Hygro] from Promega, Madison, WI, USA). We used NanofectinTM (PAA) for transfection according to the supplier's instructions. Cells were treated 24 h after transfection with pyocyanin (100 *μ*M) for 4 h. Subsequently the NF-*κ*B activity was measured by Dual-Luciferase Reporter Assay System (Promega) according to the supplier's instructions.

### 2.4. qRT-PCR

RNA isolation and cDNA generation was performed as reported previously [21]. PON3 expression level was determined by quantitative real-time PCR normalized to GAPDH as described before [21]. The following Taqman primers (Eurofins, MWG Operon) were used: PON3: sense 5′-TGGGATCACAGTCTCAGCAG-3′; antisense 5′- TCCACTAAGGTGCCCAACTG- 3′; probe 5′-TGGAAAAACATGATAACTGGGA-3′; GAPDH: sense 5′-CAACAGCCTCAAGATCATCAGC-3′; antisense 5′-TGGCATGGACTGTGGTCATGAG-3′; probe 5′-CCTGGCCAAGGTCATCCATGACAAC-3′.

### 2.5. Western Blotting

Preparation of lysates, SDS-PAGE, and Western blotting was performed as reported previously [25]. Rabbit-anti-PON3 polyclonal antibody was used at 1 : 750 (Sigma, St. Louis, MO, USA), rabbit-anti-PON2 [26] used at 1 : 2000. Mouse-anti-GAPDH 6C5 (Santa Cruz, Santa Cruz, CA, USA) and HRP-conjugated secondary antibodies were from Sigma or Cell Signaling Technology. Immunodetected proteins were visualized and quantitatively evaluated as described before [24].

ROS detection and determination of lactonase and lovastatinase activities were performed as described before [17, 24, 27].

### 2.6. Software, Statistics, and Image Acquisition

**Figure 1:**
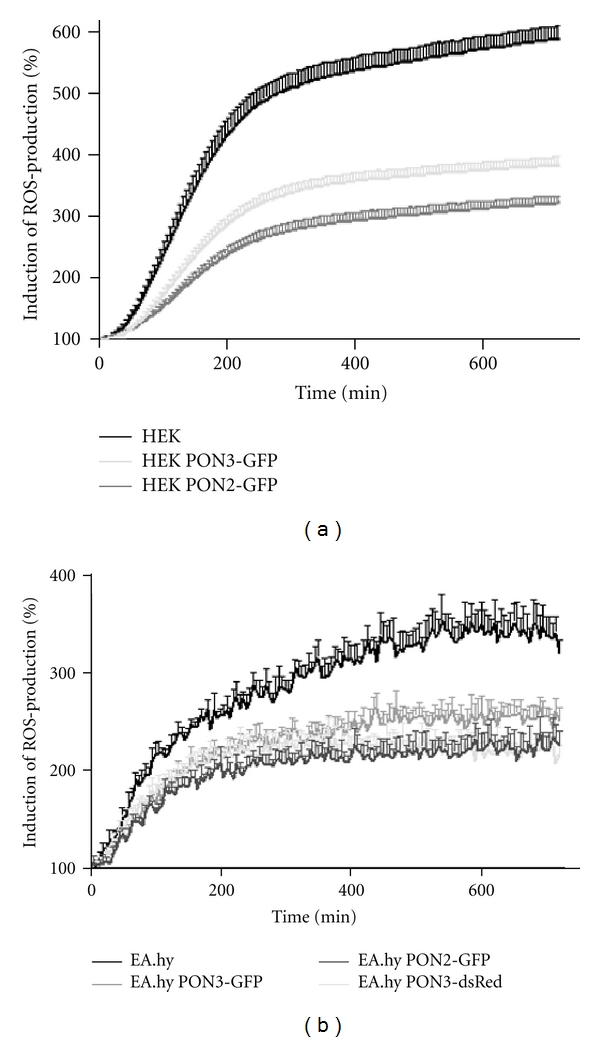
PON2 or PON3 overexpression diminishes ROS production induced by *P. aeruginosa* signaling molecule pyocyanin (PCN). (a) Naïve, PON2-GFP, or PON3-GFP overexpressing HEK293 cells were loaded with carboxy-H_2_DCFDA and stimulated with PCN (2.4 *μ*M). Carboxy-H_2_DCFDA fluorescence as means of ROS was recorded over several hours. (b) Similar to A. Naïve, PON2-GFP, PON3-GFP, or PON3-dsRed overexpressing EA.hy 926 cells were loaded with L-012 and stimulated with PCN (2.4 *μ*M). Curve maxima calculated by nonlinear regression showed statistically significant differences (*P* < 0.001) between naïve and PON2 or PON3 overexpressing cells.

**Figure 2:**
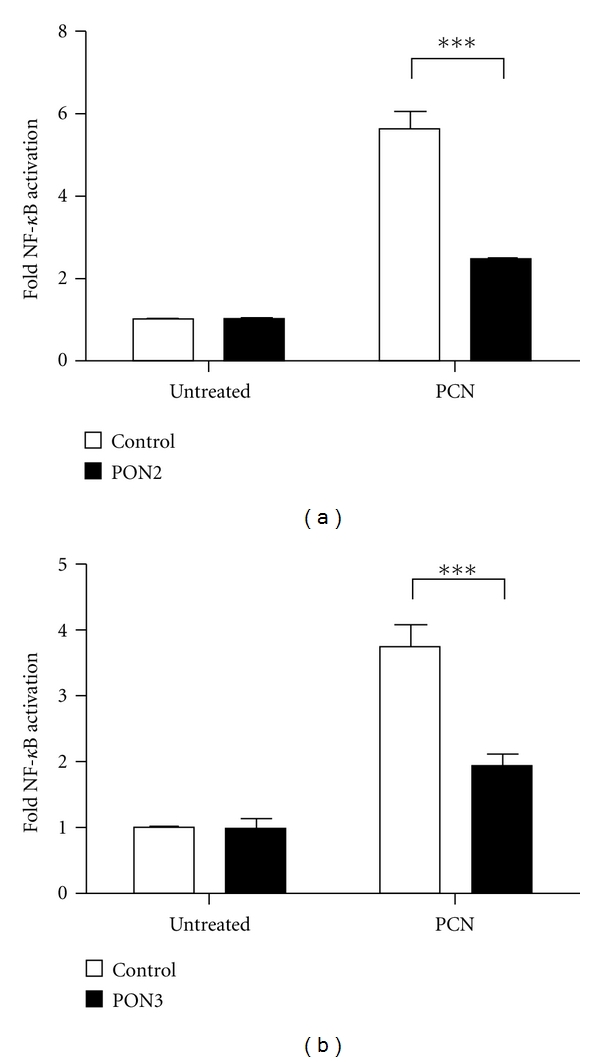
PON2 or PON3 overexpression diminishes NF-*κ*B activation induced by *P. aeruginosa* signaling molecule PCN. A549 cells transiently overexpressing HA or PON2-HA (a) and GFP or PON3-GFP (b) were stimulated with PCN (100 *μ*M, 4 h) and analyzed for NF-*κ*B activation. Symbols represent ± S.E.M. *n* = 6–9; ***P < 0.001.

**Figure 3:**
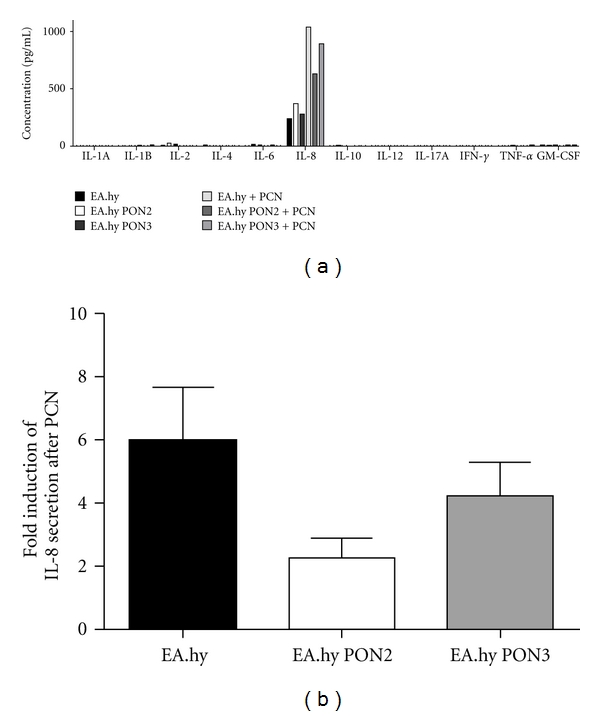
PCN induces IL-8 secretion, which can be lowered by PON2 or PON3 overexpression. (a) Naïve, PON2-GFP or PON3-GFP overexpressing EA.hy 926 cells were treated with PCN (10 *μ*M, 16 h). Cell supernatants were analyzed for the secretion of the listed cytokines and chemokines by ELISA. (b) Quantitative evaluation of results from panel (a) Fold induction of IL-8 release was calculated between untreated and PCN-treated samples.

**Figure 4:**
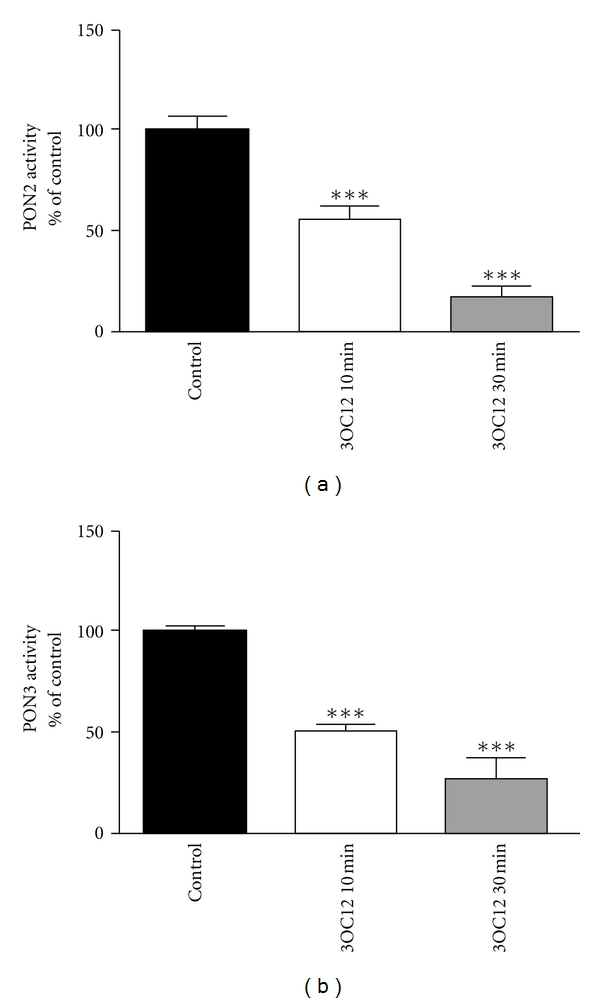
PON2 and PON3 activity decrease after 3OC12 treatment. (a) PON2-GFP overexpressing HEK293 cells were treated with 3OC12 (100 *μ*M) for the indicated durations and tested for 3OC12-HSL hydrolytic activity. (b) PON3-GFP overexpressing HEK293 cells were treated with 3OC12 (100 *μ*M) for the indicated durations and tested for lovastatinase hydrolytic activity. Symbols represent ± S.E.M. *n* = 3; ***P < 0.001.

**Figure 5:**
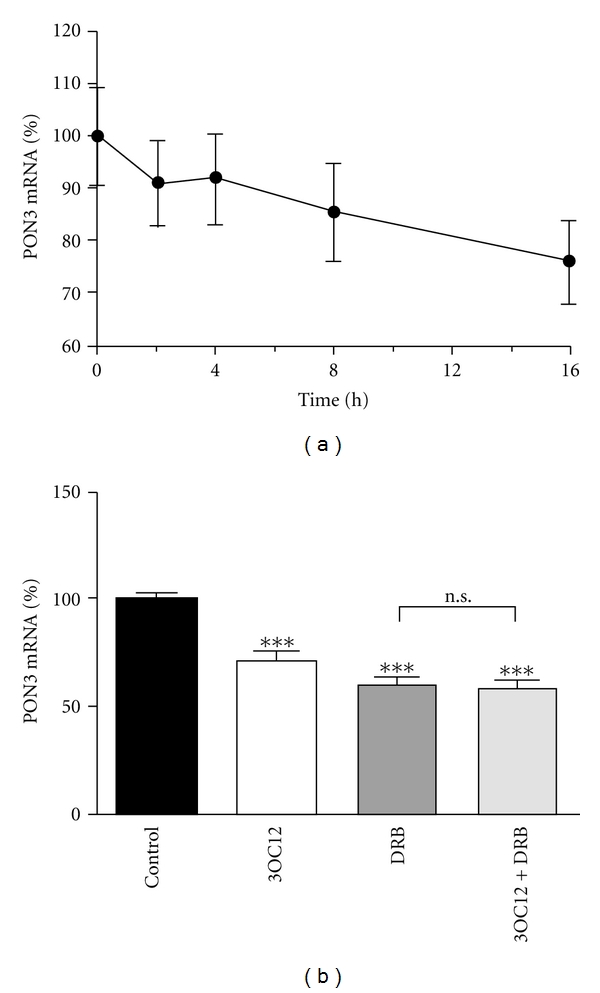
PON3 mRNA is not actively degraded in response to 3OC12 treatment. (a) A549 cells were treated with 3OC12 (100 *μ*M) for the indicated durations and analyzed for PON3 mRNA levels by qRT-PCR. (b) A549 cells were treated with 3OC12 (100 *μ*M, 24 h) or with DRB (100 *μ*M, 24 h) or combinations thereof. There was no statistically significant difference in PON3 mRNA levels after DRB or DRB/3OC12 treatment. Symbols represent ± S.E.M. *n* = 3; ***P < 0.001 versus control.

**Figure 6:**
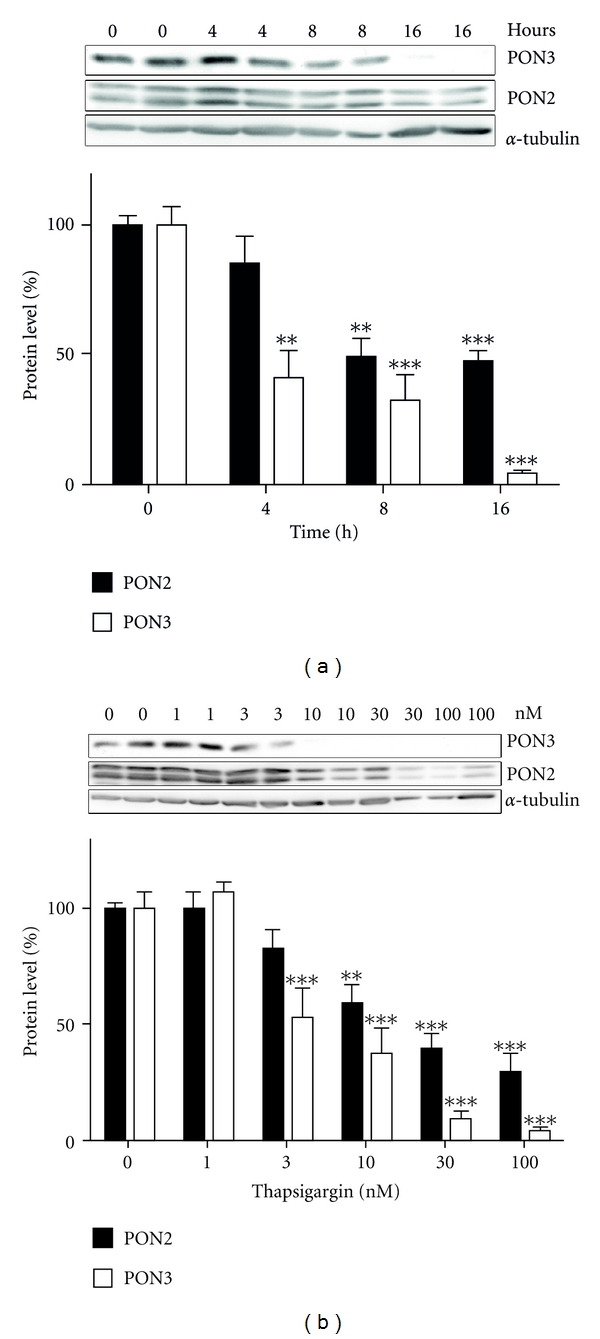
Both PON2 and PON3 are degraded after 3OC12 treatment. (a) A549 cells were treated with 3OC12 (100 *μ*M) for the indicated durations or (b) with thapsigargin (24 h) with the indicated concentrations. Lysates (50 *μ*g of protein) were analyzed by Western blotting using anti-PON2, anti-PON3, or anti-*α*-tubulin antibodies. One representative blot is shown. Results (right) are the means ± S.E.M. of three replicate analyses; **P < 0.01; ***P < 0.001.

GraphPad Prism-5 was used for calculations, statistical evaluation using 1-/2-way ANOVA with Bonferroni's multiple comparisons posttest (see Figures 1–6). *P* < 0.05 was considered significant. Adobe Photoshop software was used for image acquisition. If necessary, only brightness and/or contrast were changed simultaneously for all areas of any blot.

## 3. Results

### 3.1. PON2 and PON3 Protect Cells from PCN-Induced ROS and Inflammatory Responses

 PCN is essential for *P. aeruginosa* infections *in vivo* and causes oxidative stress, which leads to serious damage of airway epithelial cells [12, 13]. We recently found that PON2 decreases ROS production in EA.hy 926 cells in response to treatment with PCN [24]. Given the high degree of homology between the paraoxonases and previous descriptions of PON3 anti-oxidative effects, we wanted to test whether PON3 also attenuated PCN-induced ROS production. This was measured by loading naïve or PON3 overexpressing cells with the fluorescent ROS indicator carboxy-H_2_DCFDA followed by stimulation with PCN (2.4 *μ*M); PON2 overexpressing cells were used in these (and subsequent) studies for comparison. As with PON2, PON3 overexpression afforded a marked protection against PCN-induced oxidative stress (Figure 1(a)). To control for a potential direct oxidation of carboxy-H_2_DCFDA by PCN, an unwanted effect by the GFP tag and for effects specific to HEK293 cells, we also used the luminol derivate L-012 to report ROS in EA.hy 926 cells overexpressing a PON3-dsRed construct. As with HEK293 PON3-GFP cells, the EA.hy 926 PON3-GFP and PON3-dsRed cells show decreased ROS production after PCN treatment (Figure 1(b)).

 PCN also leads to a proinflammatory response by causing the release of interleukins, which might be triggered by activation of NF-*κ*B [12, 28]. To test for anti-inflammatory effects of PON2 and PON3, we next addressed activation of the NF-*κ*B pathway in lung epithelial A549 cells in response to PCN treatment. Employing gene reporter studies, we cotransfected A549 cells with (i) an NF-*κ*B firefly luciferase reporter plasmid, (ii) a renilla luciferase expression vector for normalization purposes, and (iii) a PON2 or PON3 expression plasmid. Controls received the same vectors, but without PON2/PON3 inserts. As expected, NF-*κ*B promoter activity was significantly increased 4- to 6-fold above control cells following PCN treatment (Figure 2). Importantly, PON2 and PON3 overexpression caused a dramatic decrease in activation of this major pathway involved in inflammatory response.

 Finally, because NF-*κ*B has both pro- and anti-inflammatory properties, we wished to determine which cytokine is secreted from endothelial cells exposed to PCN and if this was altered by paraoxonase overexpression. For this purpose, we performed an ELISA with a panel of inflammatory cytokines by using cell supernatants from PCN-treated naïve EA.hy 926 cells or cells overexpressing PON2-GFP or PON3-GFP. By using this approach, we covered several major inflammatory mediators, like IL-1A, IL-1B, IL-2, IL-4, IL-6, IL-8, IL-10, IL-12, IL-17A, IFN-*γ*, TNF-*α*, and GM-CSF. Remarkably, only one single cytokine was induced by PCN in endothelial cells, namely, IL-8 (>1000 pg/mL); the IL-8 level was about 6-fold higher in PCN treated compared to untreated EA.hy 926 cells (Figure 3(a)). IL-8 release was markedly lowered by PON2 or by PON3 overexpression (about 2-fold to 4-fold, respectively; Figure 3(b)). Taken together, PON2 and PON3 act as potent anti-inflammatory enzymes, which is shown by their reducing effects on ROS production, NF-*κ*B activation and IL-8 release in response to *P. aeruginosa* virulence factor PCN.

### 3.2. 3OC12 Downregulates PON3 Hydrolytic Activity and Protein

 Besides the virulence factor PCN, *P. aeruginosa* secrets the quorum sensing signal 3OC12, which can be hydrolyzed and thus inactivated by PON2. In fact, it appears that PON2 has a dominant role in 3OC12 hydrolysis [27]. Our further studies showed that PON2 hydrolytic activity, mRNA, and protein are actively downregulated by 3OC12, which disrupts PON2's protection against both 3OC12 levels and PCN-induced ROS production [24]. Given that PON3 diminished PCN-triggered ROS, it is worthwhile to address effects of 3OC12 on PON3 in a similar manner. To this end, we addressed the effect of 3OC12 on PON3 hydrolytic activity, mRNA, and protein. We used PON2-GFP or PON3-GFP overexpressing HEK293 cells for measuring lactonase or lovastatinase activity (for PON2 and PON3, resp.). In agreement with previous observations in other cell lines, PON2 activity was rapidly decreased after 3OC12 treatment. The PON2 hydrolytic activity was reduced by ~50% in 10 min and >80% in 30 min (Figure 4(a)). Our previous studies revealed that PON2 activity was inversely related to intracellular Ca^2+^-homeostasis, with 3OC12 hydrolysis being depleted in response to Ca^2+^-release as it was inhibitable by Ca^2+^-chelator BAPTA [24]. Interestingly, PON3 lovastatinase activity was also rapidly inactivated by 3OC12 to an almost identical level as observed for PON2 (Figure 4(b)).

 Our previous data demonstrated that 3OC12 caused a pronounced calcium influx in A549 and EA.hy 926 cells in a very short time-interval. This formed the basis for an active, calcium-dependent inactivation and subsequent degradation of PON2. Therefore, we wanted to explore if PON3 is also degraded in a calcium-sensitive manner, as this could point to regulatory pathways shared by these two different enzymes [24]. To this end, we treated A549 cells with 3OC12 and analyzed PON3 mRNA levels by qRT-PCR at different time-points.  Figure 5(a) shows a decrease of PON3 mRNA after 16 h 3OC12 treatment to ~70%. Next we verified, if PON3 mRNA is actively degraded or if the decrease results from a discontinued transcription or reflection of normal mRNA turnover. We treated A549 cells with 3OC12 or the RNA synthesis inhibitor 5,6-dichlorobenzimidazole 1-*β*-D-ribofuranoside (DRB), or combinations thereof. Unlike PON2, PON3 mRNA was not actively degraded in response to 3OC12 (Figure 5(b)).

 Finally we performed Western blot analyses to monitor PON3 protein levels after major Ca^2+^-disturbances caused by treatment with 3OC12. For reasons of comparison, we used the SERCA inhibitor thapsigargin, which also causes serious Ca^2+^-disturbances. Treatment of A549 cells with 3OC12 for different durations showed that PON3 protein level decreased time dependently and vanished nearly completely after 16 h; while PON2 protein was also degraded by ~50% after 16 h (Figure 6(a)). In accordance, A549 cells treated with different thapsigargin concentrations showed a significant dose-dependent degradation of PON3 and PON2 (Figure 6(b)).

## 4. Discussion

*P. aeruginosa* infections are difficult to treat since the bacteria often develop multiple antibiotic resistance and form a biofilm, which hinders the access of the antibiotics to the bacteria. Additionally, *P. aeruginosa* secrets different virulence factors, which regulate the bacterial communication and damage the infected host. Therefore, it is important to understand *P. aeruginosa *host-pathogen interactions to identify new potential therapeutic targets. Combined with the knowledge gained in previous studies, our results suggest that human paraoxonases PON2 and PON3 comprise a major defense against virulence factor-induced oxidative stress, inflammatory response, and cytokine release.

 In this study, we revealed, for the first time, the protective effect of PON3 against PCN-induced host cell damage. By various technical approaches and using different cell lines, we showed that PCN leads to ROS production, NF-*κ*B-activation, and IL-8 secretion, which can be prevented by PON2 or PON3 overexpression. PCN induces the production of O_2_^−^ and H_2_O_2_, which causes damage in various cell types [10]. Overexpression of PON2 or PON3 in relevant systems leads to a significant reduction of ROS production, reflecting the protective effect of both enzymes against PCN-induced oxidative damage. Importantly, PON2 and PON3 differ in their substrate specificities, as PON2 has a dominant lactonase activity, whereas PON3 has much better activity with some large lactones or arylesters (i.e., statins; estradiol acetates). Thus, we conclude that PON2 and PON3 act by a common anti-oxidative mechanism. Intriguingly, our data also suggest that the anti-oxidative effect of PON3 is independent from its enzymatic activity. This would be in agreement with previous results demonstrating an independent anti-oxidative and hydrolytic activity of PON2 [22]. Addressing the underlying anti-oxidative mechanism of PON2 and PON3, we and others recently showed that PON2 and PON3 localize to the inner mitochondrial membrane where they interact with coenzyme Q10 (coQ10) resulting in abrogated superoxide production [16, 22, 23]. According to the current model (reviewed in this issue; [29]), it is assumed that PON2 and PON3 protect against ROS formation by acting as an insulator for coQ10 to prevent coincidental superoxide production at the mitochondrial membrane.

 It has also been reported that PCN increases IL-8 expression in human airway epithelial cells [28]. Similarly, we observed an increase in IL-8 secretion by PCN in endothelial EA.hy 926 cells. Interestingly, this can be lowered by PON2 or PON3. Additionally, we monitored the release of numerous proinflammatory cytokines and chemokines after PCN treatment. None of the tested factors was induced except for IL-8, suggesting that IL-8 acts as a central mediator of endothelial inflammation triggered by PCN. It is known that IL-8 promoter activity and expression can be induced by NF-*κ*B [30, 31]. Our data also imply a role for NF-*κ*B as mediator between PCN-induced effects on ROS formation and proinflammatory immune response by release of IL-8. In particular, PCN was able to activate NF-*κ*B, which could be reduced by PON2 or PON3. It is fully established that NF-*κ*B is regulated by redox signaling. Taken together, our data suggest that PON2 and PON3 anti-inflammatory activities result from their ability to prevent ROS formation, as less oxidative stress likely diminishes NF-*κ*B activation and subsequent IL-8 release.

 Furthermore, we revealed the effect of 3OC12 on PON3 hydrolytic activity, mRNA, and protein. PON3 mRNA, in contrast to PON2 mRNA [22], was not actively degraded, indicative of independent mechanisms that regulate stability of these two mRNAs. PON3 protein levels were dramatically decreased by 3OC12 treatment. A severely altered calcium homeostasis as underlying mechanism appears highly likely, as this has previously been demonstrated for PON2 [24]. Our data may also suggest that PON2 and PON3 degradation occurs through the same Ca^2+^-mediated pathway, as it acts in a similar time (in case of 3OC12) or dose-dependent (in case of thapsigargin) manner. Similar to PON2, PON3 hydrolytic activity was decreased much more extensively and rapidly than the protein, indicating a likely posttranslational event blocking PON2's and PON3's enzymatic function.

 Our findings emphasize roles for PON2 and PON3 in the defense against *P. aeruginosa *virulence but show also that the bacterium may circumvent the protection by PON2 and PON3. Identification of the posttranslational modification of PON2, which causes its inactivation and induces the signaling pathway that mediates PON2 and PON3 downregulation, may lead to the identification of an important therapeutic approach. It may be beneficial to block the 3OC12-mediated decrease of PON2 activity, sustaining PON2's protective effect of inactivating 3OC12. Given the high similarity of PON2 and PON3 and considering the fact that both proteins are inactivated by 3OC12-mediated Ca^2+^-disturbances, the same regulatory posttranslational modification may reside in conserved position(s). The distinct modification remains to be determined, as protein function can be altered by many modifications, like methylation, acetylation, ubiquitinylation, and glycosylation/deglycosylation. Our previous data showed that PON2 inactivation is rapid and reversible, which would be consistent with phosphorylation/dephosphorylation by protein kinases/phosphatases [24]. If true, about 50 potential serine, threonine, and tyrosine phosphorylation residues could be responsible for the (in-)activation of PON2. Hence, future studies are needed to identify the precise mechanism that regulates enzymatic activity of PON2 and PON3. Given that inactivation occurs within minutes and the knowledge that activity does not require cofactors (except for calcium), the presence of a Ca^2+^-triggered, regulatory posttranslational modification appears highly likely. Revealing this mechanism is of great interest, not only for protection against *P. aeruginosa* virulence factors but also for activities of paraoxonases beyond this specific interaction.

## Abbreviations

carboxy-H_2_DCFDA: 5-(and-6)-carboxy-2′, 7′-dichlorodihydrofluorescein diacetate
coQ10: coenzyme Q10
IL-8: Interleukin-8
PCN: Pyocyanin
PON: Paraoxonase
ROS: Reactive oxygen species
3OC12: *N*-(3-oxododecanoyl)-L-homoserine lactone.

## References

[1] Driscoll JA, Brody SL, Kollef MH (2007). The epidemiology, pathogenesis and treatment of *Pseudomonas aeruginosa* infections. *Drugs*.

[2] Boontham P, Robins A, Chandran P (2008). Significant immunomodulatory effects of *Pseudomonas aeruginosa* quorum-sensing signal molecules: possible link in human sepsis. *Clinical Science*.

[3] Smith RS, Fedyk ER, Springer TA, Mukaida N, Iglewski BH, Phipps RP (2001). IL-8 production in human lung fibroblasts and epithelial cells activated by the Pseudomonas autoinducer N-3-oxododecanoyl homoserine lactone is transcriptionally regulated by NF-*κ*B and activator protein-2. *Journal of Immunology*.

[4] Li H, Wang L, Ye L (2009). Influence of *Pseudomonas aeruginosa* quorum sensing signal molecule N-(3-oxododecanoyl) homoserine lactone on mast cells. *Medical Microbiology and Immunology*.

[5] Jacobi CA, Schiffner F, Henkel M (2009). Effects of bacterial N-acyl homoserine lactones on human Jurkat T lymphocytes-OdDHL induces apoptosis via the mitochondrial pathway. *International Journal of Medical Microbiology*.

[6] Smith RS, Harris SG, Phipps R, Iglewski B (2002). The *Pseudomonas aeruginosa* quorum-sensing molecule N-(3-oxododecanoyl)homoserine lactone contributes to virulence and induces inflammation in vivo. *Journal of Bacteriology*.

[7] Bjarnsholt T, van Gennip M, Jakobsen TH, Christensen LD, Jensen PØ, Givskov M (2010). In vitro screens for quorum sensing inhibitors and in vivo confirmation of their effect. *Nature Protocols*.

[8] Hentzer M, Wu H, Andersen JB (2003). Attenuation of *Pseudomonas aeruginosa* virulence by quorum sensing inhibitors. *The EMBO Journal*.

[9] Lau GW, Hassett DJ, Ran H, Kong F (2004). The role of pyocyanin in *Pseudomonas aeruginosa* infection. *Trends in Molecular Medicine*.

[10] Hassan HM, Fridovich I (1980). Mechanism of the antibiotic action of pyocyanine. *Journal of Bacteriology*.

[11] Reszka KJ, O’Malley Y, McCormick ML, Denning GM, Britigan BE (2004). Oxidation of pyocyanin, a cytotoxic product from *Pseudomonas aeruginosa*, by microperoxidase 11 and hydrogen peroxide. *Free Radical Biology and Medicine*.

[12] Rada B, Lekstrom K, Damian S, Dupuy C, Leto TL (2008). The Pseudomonas toxin pyocyanin inhibits the dual oxidase-based antimicrobial system as it imposes oxidative stress on airway epithelial cells. *Journal of Immunology*.

[13] Lau GW, Ran H, Kong F, Hassett DJ, Mavrodi D (2004). *Pseudomonas aeruginosa* pyocyanin is critical for lung infection in mice. *Infection and Immunity*.

[14] Primo-Parmo SL, Sorenson RC, Teiber J, La Du BN (1996). The human serum paraoxonase/arylesterase gene (PON1) is one member of a multigene family. *Genomics*.

[15] Marsillach J, Mackness B, Mackness M (2008). Immunohistochemical analysis of paraoxonases-1, 2, and 3 expression in normal mouse tissues. *Free Radical Biology & Medicine*.

[16] Schweikert E-M, Devarajan A, Witte I PON3 is upregulated in cancer tissues and protects against mitochondrial superoxide-mediated cell death.

[17] Draganov DI, Teiber JF, Speelman A, Osawa Y, Sunahara R, La Du BN (2005). Human paraoxonases (PON1, PON2, and PON3) are lactonases with overlapping and distinct substrate specificities. *Journal of Lipid Research*.

[18] Teiber JF, Billecke SS, La Du BN, Draganov DI (2007). Estrogen esters as substrates for human paraoxonases. *Archives of Biochemistry and Biophysics*.

[19] Draganov DI (2010). Lactonases with oragnophosphatase activity: structural and evolutionary perspectives. *Chemico-Biological Interactions*.

[20] Stoltz DA, Ozer EA, Ng CJ (2007). Paraoxonase-2 deficiency enhances *Pseudomonas aeruginosa* quorum sensing in murine tracheal epithelia. *American Journal of Physiology*.

[21] Witte I, Altenhöfer S, Wilgenbus P (2011). Beyond reduction of atherosclerosis: PON2 provides apoptosis resistance and stabilizes tumor cells. *Cell Death and Disease*.

[22] Altenhöfer S, Witte I, Teiber JF (2010). One enzyme, two functions: PON2 prevents mitochondrial superoxide formation and apoptosis independent from its lactonase activity. *Journal of Biological Chemistry*.

[23] Devarajan A, Bourquard N, Hama S (2011). Paraoxonase 2 deficiency alters mitochondrial function and exacerbates the development of atherosclerosis. *Antioxidants and Redox Signaling*.

[24] Horke S, Witte I, Altenhöfer S (2010). Paraoxonase 2 is down-regulated by the *Pseudomonas aeruginosa* quorum-sensing signal N-(3-oxododecanoyl)-L-homoserine lactone and attenuates oxidative stress induced by pyocyanin. *Biochemical Journal*.

[25] Horke S, Witte I, Wilgenbus P (2008). Protective effect of paraoxonase-2 against endoplasmic reticulum stress-induced apoptosis is lost upon disturbance of calcium homoeostasis. *Biochemical Journal*.

[26] Horke S, Witte I, Wilgenbus P, Krüger M, Strand D, Förstermann U (2007). Paraoxonase-2 reduces oxidative stress in vascular cells and decreases endoplasmic reticulum stress-induced caspase activation. *Circulation*.

[27] Teiber JF, Horke S, Haines DC (2008). Dominant role of paraoxonases in inactivation of the *Pseudomonas aeruginosa* quorum-sensing signal N-(3-oxododecanoyl)-L-homoserine lactone. *Infection and Immunity*.

[28] Denning GM, Wollenwebber LA, Railsback MA, Cox CD, Stoll LL, Britigan BE (1998). Pseudomonas pyocyanin increases interleukin-8 expression by human airway epithelial cells. *Infection and Immunity*.

[29] Witte I, Foerstermann U, Devarajan A, Reddy S, Horke S Protectors and traitors—the roles of PON2 and PON3 in atherosclerosis and cancer.

[30] Kunsch C, Rosen CA (1993). NF-*κ*B subunit-specific regulation of the interleukin-8 promoter. *Molecular and Cellular Biology*.

[31] Kang HB, Kim YE, Kwon HJ, Sok DE, Lee Y (2007). Enhancement of NF-*κ*B expression and activity upon differentiation of human embryonic stem cell line SNUhES3. *Stem Cells and Development*.

